# The Brain That Understands Diversity: A Pilot Study Focusing on the Triple Network

**DOI:** 10.3390/brainsci15030233

**Published:** 2025-02-23

**Authors:** Taiko Otsuka, Keisuke Kokubun, Maya Okamoto, Yoshinori Yamakawa

**Affiliations:** 1 Graduate School of Management, Kyoto University, Kyoto 606-8501, Japan; 2 Institute of Innovative Research, Tokyo Institute of Technology, Meguro, Tokyo 152-8550, Japan; 3 ImPACT Program of Council for Science, Technology and Innovation (Cabinet Office, Government of Japan), Chiyoda, Tokyo 100-8914, Japan; 4 Office for Academic and Industrial Innovation, Kobe University, Kobe 657-8501, Japan; 5 Brain Impact, Kyoto 606-8501, Japan

**Keywords:** diversity, gender, gray matter volume, LGBTQ, origin, triple network

## Abstract

**Background/Objectives**: Interest in diversity is growing worldwide. Today, an understanding and social acceptance of diverse people is becoming increasingly important. Therefore, in this study, we aimed to clarify the relationship between an individual’s gray matter volume (GMV), which is thought to reflect brain health, and their understanding of diversity (gender, sexuality (LGBTQ), and origin). **Methods**: GMV was determined as the value of the Gray Matter Brain Healthcare Quotient (GM-BHQ) based on MRI image analysis. Meanwhile, participants’ understanding and acceptance of diversity was calculated based on their answers to the psychological questions included in the World Values Survey Wave 7 (WVS7). **Results**: Our analysis indicated that, in the group of participants with the highest understanding of diversity (PHUD. n = 11), not only the GMV at the whole brain level (t = 2.587, *p* = 0.027, Cohen’s d = 0.780) but also the GMV of the central executive network (CEN: t = 2.700, *p*= 0.022, Cohen’s d = 0.814) and saliency network (SN: t = 3.100, *p* = 0.011, Cohen’s d = 0.935) were shown to be significantly higher than the theoretical value estimated from sex, age, and BMI at the 5% level. In addition, the GMV of the default mode network (DMN: t = 2.063, *p* = 0.066, Cohen’s d = 0.622) was also higher than the theoretical value at the 10% level. Meanwhile, in the group of others (n = 10), there was no significant difference from the theoretical value. These differences between PHUD and others were also observed when comparing the two with and without controlling for educational and occupational covariates at the 5% or 10% levels. **Conclusions**: These results suggest that understanding diversity requires a healthy brain, centered on three networks that govern rational judgment, emotion regulation, other-awareness, self-awareness, and the valuing of actions. This is the first study to show that brain structure is related to an understanding and acceptance of the diversity of people.

## 1. Introduction

**Figure 1:**
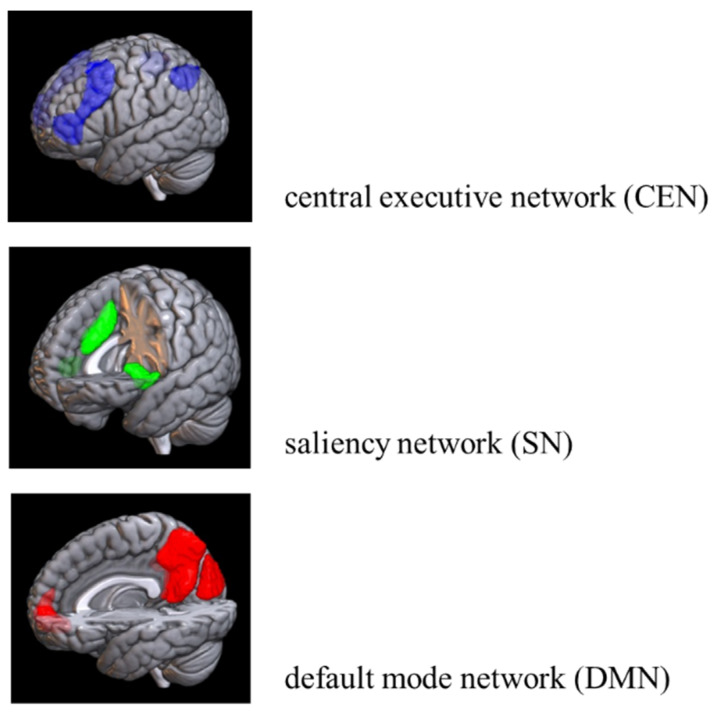
Triple network model. Retrieved from: https://prtimes.jp/main/html/rd/p/000000002.000063078.html (accessed on 21 September 2024).

As interest in diversity grows, there has been increasingly lively discussion about how we can accept the diversity of people. In the field of neuroscience, there is active research on the differences in brain structure and function between men and women, LGBTQ individuals, and those of different origins [1,2,3,4]. On the other hand, there has been little research into what the brains of people who accept such diversity are like. However, to realize a society in which women, LGBTQ people, and people of different origins can thrive, an understanding of, and compassion for, diversity is necessary. Compassion is said to be an individual’s ability to empathize with the suffering of others [5]. Therefore, it is not impossible to hypothesize and analyze the relationship between brain health, which is the state of brain functioning across cognitive, sensory, social-emotional, behavioral, and motor domains, allowing a person to realize their full potential over their life course, irrespective of the presence or absence of disorders [6], and an understanding of diversity by referring to the mechanism of empathy, the ability to share someone else’s feelings or experiences by imagining what it would be like to be in that person’s situation [7], that has been dealt with in the field of neuroscience. Here, the triple network model [8], which consists of three networks—the saliency network (SN), the central executive network (CEN), and the default mode network (DMN)—is essential for dealing with empathy. See Figure 1 for the location of each network.

Of these, the CEN, composed of the dorsolateral prefrontal cortex and the posterior parietal cortex, is important for the active retention and manipulation of information in working memory, attention, problem-solving, decision-making, and self-awareness [9,10,11,12,13,14,15]. The SN is also a network that includes the ventrolateral prefrontal cortex (VLPFC) and the anterior insula (collectively referred to as the frontal insular cortex FIC) and the anterior cingulate cortex (ACC) [16], responding to subjective degrees of salience, whether cognitive, homeostatic, or emotional [17]. The SN also acts as a switch between the CEN and the DMN, inhibiting the latter and activating the former when a salient stimulus or cognitive task is at hand, a process essential for attention and flexible cognitive control [18,19,20,21,22,23]. On the other hand, the DMN includes the medial posterior cortex, including the posterior cingulate cortex (PCC) and part of the frontal bone, the medial prefrontal cortex (MPFC), and the posterior temporal region around the temporoparietal junction (TPJ), including the inferior parietal lobule (IPL) [24,25,26]. The DMN is preferentially activated when the individual is not focused on the external environment [25] and is involved in various areas of cognitive and social processing. That is, the medial prefrontal cortex (MPFC) plays an important role in the social understanding of others, and the connections between the anterior MPFC (aMPFC) and the posterior and anterior cingulate cortices primarily contribute to self–other discrimination. On the other hand, the relationship between the dorsal MPFC (dMPFC) and the temporoparietal junction (TPJ) is mainly related to understanding the mental state of others [27].

Interactions between these networks are related to everyday self-regulation and empathy for others [28,29,30,31,32,33]. However, to the best of our knowledge, no research has dealt with the relationship between brain structure and function centered on these triple networks and the understanding of diversity along the lines of gender, sexuality, and origin. Therefore, in this study, we will contribute to the development of diversity research by clarifying the characteristics of the brain’s structure, centered on the triple network, of people who accept diversity.

## 2. Materials and Methods

### 2.1. Participants

In this study, GMV values and diversity acceptance were obtained to clarify the brain structure of people who accept diversity. GMV values were obtained from the GM-BHQ based on MRI image analysis. Diversity acceptance attitudes were obtained from a questionnaire survey using items included in the WVS7. The MRI images were obtained at the Tokyo Institute of Technology from October to November 2022, and responses to the questionnaire were obtained online from April to May 2023. The sample size required to perform a *t* test on deviations from a constant value equivalent to an effect size of 0.8, which corresponds to a “large” effect size according to Cohen [34], a significance level of 10%, and a power of 80%, was calculated as 12 samples using G*Power 3.1.9.7. We set a large effect size because a review paper on emotions-focused mindfulness-related interventions reported large effect sizes > 0.8 in the whole brain’s gray matter [35]. We also set a large *p*-value because the topic we dealt with is novel and we wanted to liberally accept the risk of type I error. For example, in a pilot study on preventing complications in patients with traumatic brain injury, statistical significance was defined with a *p* value of <0.05 while non statistical significance was defined with a *p* value of >0.1 [36]. 

A total of 22 people (20 men and 2 women) participated in both the MRI image acquisition and the questionnaire information collection process, with a mean age of 47.6 ± 12.7 years. Of the 22 participants, 1 had type 1 diabetes. Because several previous studies have shown an association between diabetes and brain atrophy [37,38], the data obtained for this one subject were excluded. As a result, the final number of participants was 21 people (19 men and 2 women) with a mean age of 47.2 ± 12.8 years. A total of 113 people (91 men and 22 women) with a mean age of 44.8 ± 11.8 years, including these 21 people, participated in obtaining MRI images to be used to calculate theoretical GM-BHQ values controlled for sex, age, and BMI. It should be noted that the 21 people were those who responded to a call to participate in a diversity survey. This type of sampling is prone to bias, with many participants being highly interested in diversity. To control for this bias, a group of participants with the highest understanding of diversity (PHUD) was defined and extracted based on the absolute value of the responses, as described later. All methods were carried out in accordance with the relevant guidelines and regulations, all participants gave written informed consent before participating, and their anonymity was maintained. The study was conducted with the approval of the Tokyo Institute of Technology’s ethical committee for “Brain information cloud (research ethics review committee for human subjects: permission number 2019007)”.

### 2.2. Questionnaire Items

Gender-, LGBTQ-, and origin-related questions from the World Value Survey WAVE7 [39], which collects information on the values and beliefs of people around the world, were extracted. The use of these globally used items is intended to facilitate interpretation of our results in this study and to inform future cross-disciplinary research. Below are the three questions that were selected:

(i) Gender: Please tell me how essential you think it is as a characteristic of democracy.

“Women have the same rights as men.” (Q249)

(ii) LGBTQ: Could you please mention any individuals that you would not like to have as neighbors?

“Homosexual” (Q22)

(iii) Different origin: Could you please mention any individuals that you would not like to have as neighbors?

“People of a different race” (Q19)

Question (i) above has 10 answers ranging from “1: Not an essential characteristic of democracy” to “10: An essential characteristic of democracy”, and questions (ii) and (iii) have 6 answers ranging from “1: I don’t want to live in the neighborhood” to “6: I can live in the neighborhood”, both on a Likert scale.

### 2.3. Calculation of ΔGMV

GM-BHQ was used to calculate GMV. GM-BHQ is a standardized index of the brain’s gray matter volume calculated from T1-weighted images with an average of 100 and a standard deviation of 15. It has been approved as an international standard by the standardization organization ITU-T as a “numerical index representing the physical characteristics of the brain that indicate health-related conditions” (approval number: ITU-TH.861.0). In previous research, GM-BHQ at the whole-brain level was positively correlated with curiosity [40], empathic concern [41], and cognitive ability [42], and negatively correlated with stress [43], unbalanced diet [44], and unhealthy lifestyle [45]. See Nemoto et al. [46] for details of the GM-BHQ estimation method.

Previous studies have shown that GM-BHQ can be predicted by multiple regression using age, sex, and BMI as variables [46]. Based on this, we created a multiple regression equation with age, sex, and BMI as independent variables and GM-BHQ as dependent variables from the data of 113 people, and calculated the difference between the actual GM-BHQ value and the predicted GM-BHQ value, that is, ΔGMV, from the equation. By using ΔGMV, we analyzed the relationship between participants’ brain health and their understanding of diversity controlling for the effects of age, sex, and BMI.

### 2.4. Analytical Method

This study tested the hypothesis that people who are more inclusive about gender, LGBTQ people, and those of different origins have higher GMV. To that end, we first selected people who showed the highest degree of acceptance in the three questions shown above. Specifically, we extracted the respondents who chose “10: An essential characteristic of democracy” for question (i), and “6: I can live in the neighborhood” for question (ii) and (iii). Next, we identified respondents who selected the most positive options for all three questions as PHUD. Finally, a one-sample *t*-test to confirm whether the mean value of ΔGMV was greater than zero and an independent-sample *t*-test to confirm whether the mean value of ΔGMV was different between PHUD and others were performed. The latter, i.e., whether the mean value of ΔGMV was different between PHUD and others, was also confirmed by an F-test using an analysis of covariance (ANCOVA) that controlled for years of schooling and the occupation dummy (management = 1, others = 0). As evaluation targets, in addition to whole brain’s ΔGMV, we analyzed regional ΔGMV for CEN, SN, and DMN. Given that this was a pilot study, statistical significance was defined as a *p*-value < 0.1, although we explicitly distinguished this from the commonly used standard of a *p*-value < 0.05 following the method of previous study [36]. All statistical analyses were performed using IBM SPSS Statistics Version 28 (IBM Corp., Armonk, NY, USA).

## 3. Results

**Figure 2:**
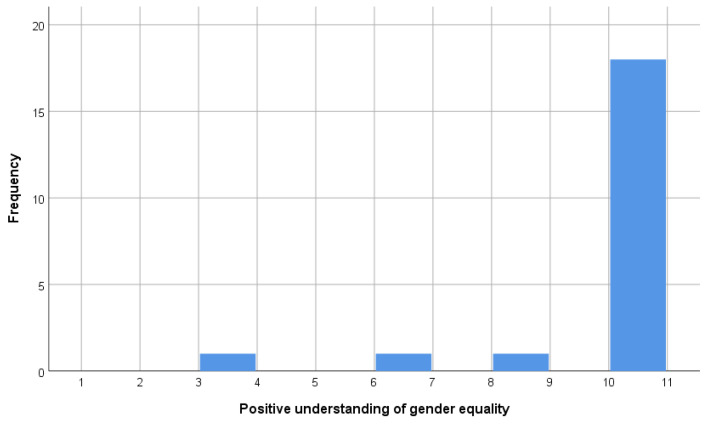
Frequency of “positive understanding of gender equality” by score.

**Figure 3:**
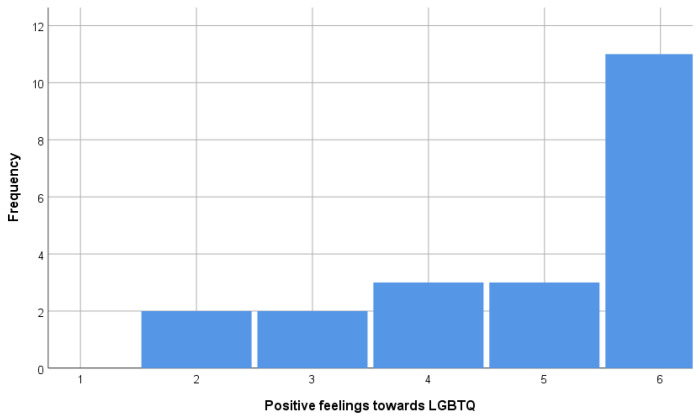
Frequency of “positive feelings towards LGBTQ” by score.

**Figure 4:**
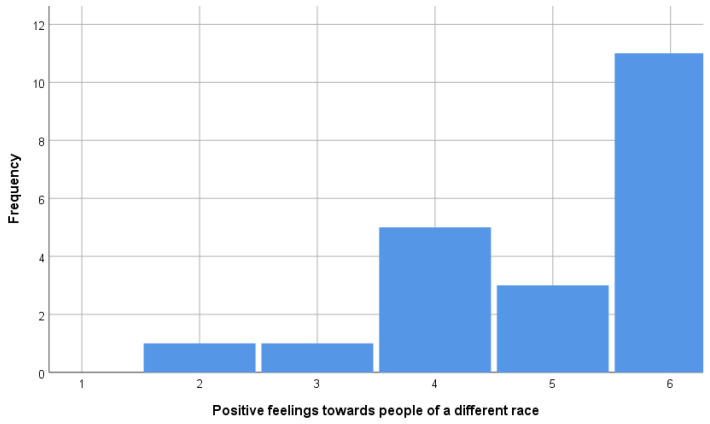
Frequency of “positive feelings towards people of a different origin” by score.

Based on the method above, PHUD (n = 11) and others (n = 10) were extracted. PHUD were identical to the respondents who answered the highest score choices in questions (ii) and (iii) and were among the 18 respondents who answered the highest score choices in question (i). The histograms showing the distribution of answers to questions (i), (ii), and (iii) are shown in Figure 2, Figure 3 and Figure 4. Table 1 and Table 2 compare the attributes of PHUD and others. There were no significant differences in the *t*-test or chi-square test for any of the items. Considering previous research showing that lifestyle [45] and socioeconomic factors [47] influence brain development, such marginal differences between groups indicate that these are good samples.

**Figure 5:**
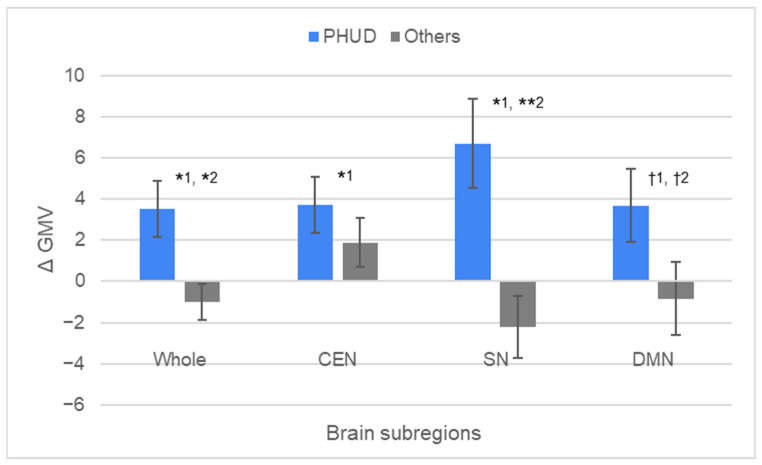
Difference in GMV from baseline by region for PHUD. ^1^ One-sample *t*-test to confirm whether the mean value of ΔGMV was greater than zero. ^2^ Independent-sample *t*-test to confirm whether the mean value of ΔGMV was different between PHUD and others. ** *p* < 0.01; * *p* < 0.05; ^†^ *p* < 0.10. PHUD: participants with the highest understanding of diversity. Error bars represent standard error.

In Figure 5, the results of a one-sample *t*-test to confirm whether the mean value of ΔGMV was greater than zero and an independent-sample *t*-test to confirm whether the mean value of ΔGMV was different between PHUD and others were indicated. Table 3 details the results of these analyses. For ΔGMV in the whole brain, PHUD had an average value of 3.544 (SD = 4.542), which was significantly higher than 0 (t = 2.587, *p* = 0.027, d = 0.780). ΔGMV by regions also showed a level significantly higher than 0 in CEN and SN, with an average value of 3.732 (SD = 4.584, t = 2.700, *p* = 0.022, d = 0.814) and 6.712 (SD = 7.181, t = 3.100, *p* = 0.011, d = 0.935), respectively. The mean value of DMN was 3.697 (SD = 5.943, t = 2.063, *p* = 0.066, d = 0.622), so the difference between ΔGMV and 0 was significant at the 10% level. In contrast, the results for “others” were all not significantly different from the theoretical values. The PHUD’s ΔGMV was generally higher than that of others: GMV in the whole brain (t = 2.728, *p* = 0.013, d = 1.206), CEN (t = 0.994, *p* = 0.333, d = 0.436), SN (t = 3.308, *p* = 0.004, d = 1.460), and DMN (t = 1.795, *p* = 0.089, d = 0.786). These results were replicated in ANCOVA’s F-test controlling for years of schooling and occupation dummies: GMV in the whole brain (F = 6.625, *p* = 0.020, η^2^ = 0.280), CEN (F = 1.127, *p* = 0.303, η^2^ = 0.062), SN (F = 9.321, *p* = 0.007, η^2^ = 0.354), and DMN (F = 4.732, *p* = 0.044, d = 0.218).

## 4. Discussion

In this study, people with a high level of diversity-awareness had significantly higher GMV for whole and subscale brain networks, CEN and SN, and relatively higher GMV for DMN than those estimated from age, sex, and BMI. This indicates that to accept diversity, the health of CEN, which is involved in cognitive control, is important. The CEN is a brain region involved in working memory and rational judgment, such as the dorsolateral prefrontal cortex and superior parietal lobule [9,10,11,12,13,14,15]. In addition, the analysis results showed that the health of the SN, which is responsible for external monitoring functions, and the DMN, which is closely related to sociality, are important. The SN, which includes the insular cortex, anterior cingulate gyrus, and paracingulate gyrus, is a brain region involved in emotional reactions and regulation [17,18,19,20,21,22,23]. On the other hand, the DMN includes the posterior cingulate gyrus, medial superior frontal gyrus, precuneus, angular gyrus, and infraorbital frontal lobe [24,25,26,27]. A previous study performed an analysis on resting brain activity and showed that emotional empathy scores correlated with functional connectivity in the CEN, SN, and DMN [30]. This is consistent with empathy responses in previous studies using functional magnetic resonance imaging (fMRI) [29,31,32,33], showing that emotional empathy involves movement, attention, and self-referential processing. Here, affective empathy refers to the ability to understand, infer, judge, and share the emotional experiences of others [33,48], and is important in social interactions [49,50]. As such, previous studies have shown that deficits in emotional empathy are associated with alcoholism [51] and social anxiety disorder [52].

Previous research also indicates that CEN, SN, and DMN are uniquely associated with empathy. fMRI experiments showed that both empathetic and permissive judgments activate the superior frontal gyrus that spans the CEN and DMN [53]. The superior frontal gyrus is thought to contribute to cognitive functions such as self-awareness [10] and working memory in conjunction with the action of the sensory system [54,55]. This suggests that empathy activates specific brain regions and contributes to social cohesion [55]. Similarly, studies on racial bias show that the activation of regions of the frontal cortex associated with control and regulation modulates amygdala activity, thereby suppressing racist emotions [56]. Also, some fMRI studies suggest that the lack of empathy is primarily due to a defective SN switching function. Dysfunctional SNs are involved in the activation of the DMN, which leads to self-focused attention and empathy disorders such as narcissism [57]. Furthermore, the AIs and dACCs that make up the SN have been associated with empathy themselves [58,59,60].

Alternatively, data-driven quantitative reasoning studies suggest that SN and CEN facilitate individuals to build long-term social relationships through emotional processing and cognitive control and that DMN facilitates individuals to develop long-term social relationships through mentalizing processes. It has been suggested that it predicts the experiences, beliefs, and intentions of others and facilitates interaction [61]. DMN has been implicated in both functional and structural studies as prosocial personality traits such as extraversion and agreeableness [62,63,64], and it is associated with self-cognitive empathy [65,66]. On the other hand, a recent systematic review shows that people with low compassion tend to have either low reward-related neuronal area activity or low gray matter volume [67]. Moreover, a comprehensive review of over 20 years of neuroimaging and behavioral research on the neurocognitive processes underlying humans’ ability to understand other people’s minds suggests that the coactivation of cognitive and emotional processes may be particularly relevant for ecologically valid social cognition [68]. Another large meta-analysis of neuroimaging studies has also implicated the triple network in social interactions [69]. Consistent with these, our previous studies have shown that whole-brain gray matter is positively correlated with psychological variables representing behavioral activation, empathic concern, and self-monitoring [36].

The results of the current study are consistent with this series of previous studies and at the same time, provide a compelling reason why a triple network consisting of CEN, SN, and DMN is necessary to understanding diversity. In light of the roles of the three networks, the functions they play in understanding diversity can be summarized as follows. First, DMN is required to recognize and understand people with different positions [24,25,26,27]. However, recognition and understanding alone are not enough to utilize or cooperate with them, so there is a possibility that a lasting relationship will not be built. At this time, if we can utilize CEN to pay attention to the problem, solve it, and make strategic decisions [9,10,11,12,13,14,15], it will be easier to build a rational relationship with people with different positions, and it will be easier for the side that accepts them to benefit, which may lead to a lasting relationship. Since whether DMN or CEN is required depends on the situation, a switch between the two using SN is required [18,19,20,21,22,23]. With this model in mind, let’s look back on today’s understanding of diversity in society. 

First, given that women account for half of the world’s population and that no compelling evidence has been found to show differences in work ability between men and women [70], gender role divisions of labor should distort the allocation of human resources and lead to lower productivity. A study has shown that a 10-percentage point increase in the number of female members of parliament increases gross domestic product growth by 0.74 percentage points [71]. On the other hand, LGBTQ people and people of different origins are not easily recognized as familiar in some countries and societies. Therefore, in such countries and societies, it may be difficult to make a movement to accept them. However, in a survey asking managers of companies with diversity management strategies “What are the benefits of embracing diversity?”, responses included “attracting talent”, “improving business performance”, “improving brand power and reputation”, and “stimulating innovation” [72]. This suggests that it is important for businesses and political leaders to embrace diverse perspectives not only for the purpose of contributing to the realization of a fair society, but also to strengthen the economic profit structure at the corporate and national levels. Therefore, people with a high level of understanding of diversity are thought to have an inclusive mindset, not only because they are considerate of others, but also because they can make rational judgments at the same time by empathizing with various things, thinking logically, and designing new strategies [73,74].

Previous research has attempted to clarify brain diversity [1,2,3,4] and the pathways of empathy in the brain (e.g., [9,10,11,12]). In recent years, following the concept of “neurodiversity”, advanced workplaces where people with not only developmental disorders but also LGBTQ people and other personality types are actively evaluated and given opportunities to flourish while achieving high performances have emerged [75,76]. Recent research argues that as the nature of work evolves and jobs become more specialized, organizational diversity is likely to become increasingly important and play a key role in terms of both individual employees and organizational success [77]. However, no matter how productive collaboration with diverse people may be, it is difficult to create a diverse society if the people do not have the ability to understand and empathize with them. There is a paradox in diversity: while it fosters innovation, it also sometimes brings difficult challenges to both organizations and society. Therefore, people need to have “cultural evolvability” to embrace diversity [78]. Despite this, to the authors’ knowledge, there has been no research to date that clarifies the brain characteristics necessary for understanding and empathizing with diversity. Given that gender and origin-related differences in brain and cognitive function are often discussed in an evolutionary context [47,70], it is not surprising if it is that in today’s world of globalization and the increasing need for teamwork among people of different backgrounds to perform complex tasks, people with brains that are able to understand and empathize with diversity have a better chance of survival. Culture changes the brain and cognitive functions, and changes in the brain and cognitive functions also affect culture [47]. Understanding how the brain works should increase people’s interest in methods for maintaining and treating brain health, with the aim of realizing a more diverse society. This study is the first to demonstrate that understanding diversity in areas such as gender, sexuality, and origin requires brain health centered on the triple network, and will contribute to the development of diversity research in brain science and the social sciences.

In this sense, this study is unique in that it focuses on the “structure” of the brain. Many previous studies have observed the “response” of the brain during an individual’s experience of empathy using fMRI. However, while brain responses are excellent for clarifying the mechanisms of brain networks, they do not guarantee the continuity of change. Our study used MRI to show that structural features of the brain related to the triple network may be related to diversity understanding. This finding indicates that individuals who use the triple network to improve diversity understanding may develop the related brain regions. Although the sample size of this study is 21 in total, which is certainly small for a recent study, it can be considered a valuable sample size for a pilot study, given that the sample sizes of approximately 300 MRI studies published in high-impact journals in 2017 and 2018 were 23–24 [79] and that 95.6% of the 882 MRI studies published in *Magnetic Resonance in Medicine* between 2017 and 2019 had 20 participants or less [80]. In addition, although the demographic bias of the sample in this study is certainly large, previous studies have shown that the ethnocentrism and masculinity values of Japanese people who were born and raised in a society with low diversity make it difficult to collaborate internationally with diverse talents [81,82], so the implications of this study, in which most of the sample was Japanese men, are rather significant. In other words, even in a sample of people who have been believed to have low diversity understanding, there are large individual differences in understanding of diversity, and if we consider that this is reflected in differences in brain health, the results of this study may be of great use in society, where understanding of diversity is most needed.

Finally, we will state some points to note and prospects for this study to be useful in helping people understand diversity. As previous studies have revealed, lifestyle habits are related to brain health [45]. For example, a balanced diet is important for brain health [83], but it also depends on the richness of a childhood home environment, including access to hobbies, books, and other cognitive activities, and communication with parents [84]. Furthermore, disparities in home environments are fixed by distortions in the social structure and passed down through generations [85]. Thus, ironically, many people with poor brain development and low diversity understanding may be people who have been discriminated against and oppressed by a society with low diversity understanding. Therefore, associating low diversity understanding with poor brain health due to poor lifestyle and attributing the problem to individuals’ responsibility is a case of diverting attention from the essence of the problem and putting the cart before the horse. Rather, the results of this study should be used to broadly create an environment that supports brain health and accepts diversity, by promoting support for poor families, encouraging the inclusion of diverse people in the workplace and in education, and providing mental health care to those who are oppressed or discriminated against, so that more people can maintain their brain health.

## 5. Limitations

This study has several limitations. First, the small sample size casts doubt on the robustness of the results. In particular, sampling bias due to the inclusion of only two women and the fact that all participants were Japanese limits the generalizability of the findings of this study. Additionally, the sampling method used was self-selection, which may have excluded participants with a low interest in diversity, similarly reducing the generalizability of the findings of this study. Future studies should validate this study by performing similar studies with widely selected larger samples with various genders and origins. Second, the cross-sectional analysis showed a correlation, not a causal relationship, between variables. In the future, the method of longitudinal analysis should be adopted to verify the results of this study. For example, a randomized controlled trial that measures the effects of interventions to improve brain health, such as improving lifestyle habits, on participants’ understanding of diversity, or a cohort study that periodically monitors changes in participants’ understanding of diversity after dividing them into groups based on differences in brain health, would be good ways to verify the results of this study. Third, we assessed triple networks in brain structures, but this method makes it difficult to observe fine connections in the brain. We encourage future studies to use fMRI to study the connectivity of these networks during diversity-related tasks. Finally, while the use of questions from the World Value Survey WAVE7 has the advantage of expanding the possibilities for future cross-disciplinary and international research using indicators used globally, the depth of capturing attitudes toward diversity may be limited. Future research should verify the results of this study using more comprehensive scales and clarify the relationship between subtle differences in attitudes and the brain.

## 6. Conclusions

In this study, an analysis was conducted using GMV assessed by the GM-BHQ based on MRI image analysis and results from a psychological questionnaire, and it was found that people with a high level of understanding of diversity, such as gender, LGBTQ, and different origins, had healthier brains centered on a triple network consisting of the CEN, SN, and DMN, compared to values estimated from age, sex, and BMI. This was also observed when comparing groups with high and low levels of diversity acceptance. This is the first study to suggest that the brain’s capacity to understand diversity may depend on a healthy triple network.

## Figures and Tables

**Table 1:** Attribute comparison between PHUD and others.

	PHUD		Others			
	Mean	SD	Mean	SD	t	*p*
GMV						
Whole	102.569	7.316	98.853	8.062	1.108	0.282
DMN	102.479	8.292	99.427	10.044	0.762	0.455
CEN	104.527	6.147	104.036	7.734	0.162	0.873
SN	103.576	10.605	95.884	10.319	1.681	0.109
Years of schooling	17.550	2.296	17.500	1.080	0.057	0.955
BMI	22.342	2.331	23.606	3.080	1.066	0.300
Age	50.000	12.116	44.100	13.486	1.056	0.304

CEN: central executive network. DMN: default mode network. PHUD: participants with the highest understanding of diversity. SD: standard deviation. SN: saliency network.

**Table 2:** Attribute comparison between PHUD and others (continued).

	PHUD	Others
Drinking frequency		
Every day	3	2
5–6 days a week	1	0
3–4 days a week	3	1
1–2 days a week	1	2
1–3 days a month	1	2
Seldom drink	1	1
Quitted	1	0
Don’t drink (can’t drink)	0	2
χ^2^	5.832	
*p*	0.559	
Smoking frequency		
Every day	0	3
Haven’t smoked for over a month	2	2
Don’t smoke	9	5
χ^2^	4.105	
*p*	0.128	
Marriage		
Married	10	8
Single	1	2
χ^2^	0.509	
*p*	0.476	
Occupation		
Management	4	3
Professional/technical	5	7
Sales	1	0
Service/security	1	0
χ^2^	2.434	
*p*	0.487	

PHUD: participants with the highest understanding of diversity.

**Table 3:** ΔGMV by region for PHUD and others.

					One-Sample *t*-Test			Independent-Sample *t*-Test		
	N	Mean	SD	SE	t ^1^	*p* ^1^	d ^1^	t ^2^	*p* ^2^	d ^2^
PHUD										
Whole	11	3.544	4.542	1.369	2.587	0.027 *	0.780	2.728	0.013 *	1.206
CEN	11	3.732	4.584	1.382	2.700	0.022 *	0.814	0.994	0.333	0.436
SN	11	6.712	7.181	2.165	3.100	0.011 *	0.935	3.308	0.004 **	1.460
DMN	11	3.696	5.943	1.792	2.063	0.066 ^†^	0.622	1.795	0.089 ^†^	0.786
Others								F ^3^	*p* ^3^	η ^2,3^
Whole	10	−1.011	2.811	0.889	1.137	0.285	0.360	6.625	0.020 *	0.280
CEN	10	1.896	3.791	1.199	1.582	0.148	0.500	1.127	0.303	0.062
SN	10	−2.202	4.797	1.517	1.452	0.181	0.459	9.321	0.007 **	0.354
DMN	10	−0.831	5.575	1.763	−0.472	0.648	0.149	4.732	0.044 *	0.218

^1^ A one-sample *t*-test to confirm whether the mean value of ΔGMV was greater than zero. ^2^ Independent-sample *t*-test to confirm whether the mean value of ΔGMV was different between PHUD and others. ^3^ Independent-sample F-test to confirm whether the mean value of ΔGMV was different between PHUD and others after controlling for years of schooling and occupation dummy (management = 1 and others = 0). ** *p* < 0.01; * *p* < 0.05; ^†^ *p* < 0.10. N: number of samples. PHUD: participants with the highest understanding of diversity. SD: standard deviation. SE: standard error. d: Cohen’s d.

## Data Availability

The data presented in this study are available on request from the corresponding author due to the need to protect the privacy of participants.
